# Detection of MUC1-Expressing Ovarian Cancer by C595 Monoclonal Antibody-Conjugated SPIONs Using MR Imaging

**DOI:** 10.1155/2013/609151

**Published:** 2013-09-30

**Authors:** Daryoush Shahbazi-Gahrouei, Mohammad Abdolahi

**Affiliations:** ^1^Department of Medical Physics and Medical Engineering, School of Medicine, Isfahan University of Medical Sciences, Isfahan, Iran; ^2^Department of Medical Physics and Radiation Technology, School of Paramedical Sciences, Bushehr University of Medical Sciences, Bushehr, Iran

## Abstract

The aim of this study is to find out the development and application of MUC1-expressing ovarian cancer (OVCAR3) by C595 monoclonal antibody-conjugated superparamagnetic iron oxide nanoparticles (SPIONs) using MR imaging. At the end, its use as a nanosized contrast agent MR imaging probe for ovarian cancer detection was investigated. The strategy is to use SPIONs attached to C595 mAb that binds to the MUC1, to specifically detect ovarian cancer cells. Anticancer effects and MR imaging parameters of the prepared nanoconjugate was investigated both under *in vitro* and *in vivo* experiments. The characterization of nanoconjugate includes its size, cell toxicity, flow cytometry, Prussian blue staining test and its cellular uptake as well as its biodistribution, and MR imaging was also investigated. The findings of the study showed good tumor accumulation and detection, no *in vivo* toxicity, and potential selective antiovarian cancer activity. Overall, based on the findings SPIONs-C595 nanosized probe is a selective ovarian molecular imaging modality. Further subsequent clinical trials appear warranted.

## 1. Introduction 

The high spatial resolution of MR imaging can be combined with specific MR molecular imaging agents to improve the specificity and sensitivity of cancer imaging. Magnetic nanoparticles have been used for various applications, particularly in health care, for example, immunoassay, cell separation, and molecular biology. Tumor cell targeting by the use of target-specific imaging probes is a potential strategy for molecular imaging [1–4]. Monoclonal antibodies (mAb) are among the best selective cancer MR carriers of pharmaceuticals and have proven to be valuable therapeutics for the diagnosis and treatment of cancers. One of the targets is ovarian-specific membrane antigen, MUC1, a high molecular weight transmembrane glycoprotein antigen [3–6]. Additionally, tumor marker antigen mucin-1 (MUC1) is a proposed molecular target for a novel imaging for cancer. Several studies have been showing that monoclonal antibody C595 is a useful antibody either alone or incorporation with other therapeutic methods to treat the human cancer [5, 7, 8].

In particular, superparamagnetic iron oxide nanoparticles (SPIONs) conjugated with mAb enhance contrast in MR imaging modalities. The use of antibody-conjugated MR imaging contrast agents to specifically target cancer cells has been demonstrated previously for several cancers [9–11].

In the past decades, significant approaches have been made in the development and application of MR imaging, and its role may shift from a problem-solving to a central management tool, possibly fulfilling a broad range of tasks from characterization, staging, and even early detection of ovarian cancer [12, 13].

Since many types of ovarian cancer cells express high levels of (MUC1) on their cell surface [14, 15], the imaging strategy is using SPIONs and their attachment to monoclonal antibody that binds to the MUC1 for enhancing the contrast of MUC1-expressing ovarian cancer cells. In this study, the production and evaluation of magnetic nanoprobe (SPIONs-C595) and its application as MR imaging contrast agent for targeted molecular imaging of MUC1-expressing ovarian cancer cells was investigated.

## 2. Materials and Methods

All chemical materials were prepared as described in a previous published paper by Abdolahi et al. [11]. C595 monoclonal antibody was obtained from Professor Barry J. Allen (University of New South Wales, Kogarah, NSW, Australia). Ovarian cancer cell line, OVCAR3, was purchased from National Cell Bank of Iran (Pasture Institute, Tehran, Iran).

The nanoprobe was synthesized using a three-step process as described in previous publications [11, 16, 17].

### 2.1. Characterization

Transmission electron microscopy (TEM) (Tecnai 10, FEI Company, USA), operating at 80 kV, was used to measure accurately the size distribution of particles. The samples for electron microscopy were prepared by deposition of a droplet of the nanoparticle solution onto a carbon-coated film supported on a copper grid and allowed to dry. The hydrodynamic particle size and the width of the particle size distribution (polydispersity index) of nanoparticles were obtained via photon correlation spectroscopy (PCS) using a Malvern Nano Series ZS, provided with a He/Ne laser of 633 nm wavelength.

**Figure 1:**
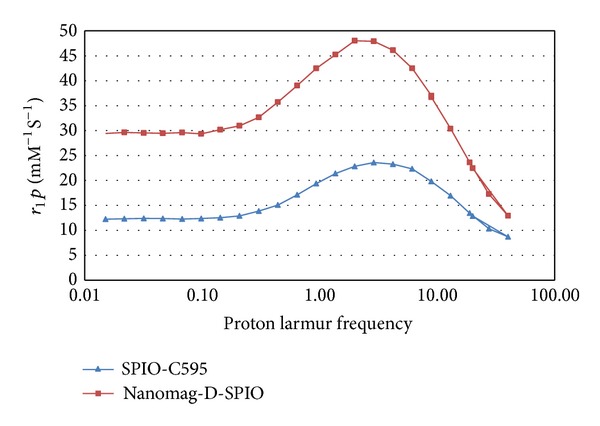
NMRD profile of Nanomag-D-SPIO and synthesized nanoparticle (SPIONs-C595) probe.

To study the magnetic properties of synthesized nanoprobe, the nuclear magnetic resonance dispersion (NMRD) profiles (the longitudinal relaxivity, *r*_1_, as a function of the magnetic field) were recorded with a field cycling relaxometer (Spinmaster FFC2000, STELAR, Italy). Additional measurements of relaxation rate (*R*_1,2_) were performed at 20 and 60 MHz and 310 K on Bruker Minispec system (Bruker, Karlsruhe, Germany) according to the inversion recovery pulse sequence and the Carr-Purcell-Meiboom-Gill pulse sequence, respectively. The results are shown in Figure 1.

The binding of mAb molecules to SPIONs and the amount of immobilized antibody were confirmed and determined by the Bradford assay method as well as the measurements of the hydrodynamic size. The iron concentration of samples was determined by the measurement of the longitudinal relaxation rate *R*_1_ at 20 MHz after digestion by microwave oven. Briefly, the samples were mineralized by microwave digestion (Milestone MLS-1200, Sorisole, Italy), and the *R*_1_ value of the resulting solutions was recorded at 0.47 T and 37°C which were also reported in published paper by Abdolahi et al. [11].

### 2.2. *In Vitro* Cytotoxicity

Human ovarian cancer (OVCAR3) cell was grown in Roswell Park Memorial Institute (RPMI-1640) medium supplemented with 10% fetal bovine serum and 1% penicillin/streptomycin followed by addition of 10 *μ*g/mL insulin. The cells were cultured in 250 mL flasks, at 37°C in a humidified atmosphere with 5% CO_2_ to allow adherence of the cells.

The cytotoxic effects of Nanomag-D-SPIO particles and the corresponding C595 mAb conjugated nanoparticles (SPIONs-C595) *in vitro* against cell lines were examined by using the 3-(4,5-dimethylthiazol-2-yl)-2,5-diphenyltetrazolium bromide (MTT) assay which is described in a previous published study [16]. All experiments were performed in triplicate, and cell survival was determined as a percentage of viable cells in comparison with controls.

### 2.3. Flow Cytometry

Flow cytometry was used to detect and quantitatively analyze cell-surface expression of MUC1 on the cell surface [17]. Briefly, cells were detached by Tripsin and washed with PBS containing 0.1% fetal bovine serum (FBS), and a 10^6^ cell per tube of each cell was transferred in FACS tubes. The cells were resuspended in 90 *μ*L of washing buffer and were preblocked with human Fc receptors blocking (human) reagent (Miltenyi) for 10 min at room temperature in the dark. After blocking, primary C595 anti MUC1 antibody (1/150 dilution) was added to each cell tube (one tube of each cell line as a control), incubated for 30 min in the dark at room temperature, and then washed 3 × 5 min using a washing buffer.

 After washing, the cells were resuspended and incubated in goat anti-mouse fluorescein isothiocyanate (FITC) mAb for an additional 30 min at room temperature in the dark. Cells were then washed, resuspended in 0.5 mL of PBS, and analyzed immediately using a CyAN-ADP flow cytometer (Beckman Coulter).

### 2.4. Cellular SPIONs Uptake Studies

The procedure for cellular iron uptake and its results for Nanomag-D-SPIO and SPIONs-C595 were described previously [11]. The potential of nanoprobes as MR imaging agent was investigated *in vitro* using 1.5 T MR imaging system with spin-echo pulse sequence as follow: *T*_*E*_ = 60 ms, *T*_*R*_ = 3000 ms, slice thickness = 2 mm, and matrix size = 512 × 512. The data from region of interest (ROI) are drawn to consistently measure mean signal intensity at the identical position within each phantom vial.

### 2.5. Prussian Blue Staining

OVCAR3 cells were detached and washed three times with PBS, and about 10^6^ cells per tube of cells were suspended in 15 mL tube and incubated with culture medium containing SPIONs-C595 at Fe concentrations of 2 mM (1 tube control) for 1 h at room temperature.

After incubation, the cells were washed three times with PBS to remove excess nanoparticles. Then, cells were fixed on 22 × 22 mm square glass coverslips with 4% glutaraldehyde, washed, and stained using specific iron Prussian blue method to observe nanoparticles accumulation. Accumulation of iron oxide nanoparticles were shown in cells as dark blue grains under microscope light using a Nikon Eclipse TS100 microscope (Nikon Corp., Tokyo, Japan).

### 2.6. Animals

The animal studies were performed with 15 nude mice, 6–8 weeks old with a mean weight of 20 g. Mice were randomly divided into three groups of five containing Nanomag-D-SPIO, synthesized nanoparticle (SPIONs-C595) and control group. Each group was housed per cage in humidity and temperature controlled and isolated animal house. All mice were fed sterilised standard mouse chow and water ad libitum.

The studied cell line (specific ovarian cancer xenograft tumors OVCAR3) was grown in tissue culture (2.5 × 10^6^ cells, 120 *μ*L) and injected subcutaneously into both flanks of nude mice. Three weeks after tumor implantation, when the tumor diameter was about 2 mm (mean weight of tumors was approximately 100 mg), mice received the amount of 0.5 mg Fe/kg (130 *μ*g Fe) intravenous (i.v.) injection of both nanoprobe MR imaging agents (SPIONs-C595, Nanomag-D-SPIO). All nanoparticle agents were diluted in physiological saline to a final concentration as injected in bolus doses.

### 2.7. *In Vivo* MR Imaging

The MR images contrast depends on the *T*_1_ and *T*_2_ relaxation times. SPIONs affect *T*_2_ and work as negative contrast agents hence decrease signal intensity. A higher intracellular concentration of SPIONs results in the reduction of *T*_2_ relaxation time. The mice were anesthetized using a nose cone that delivered an isoflurane and oxygen mixture and imaged using 1.5 T MR imaging scanner (Signa, GE Medical System, Milwaukee, WI, USA) and a standard circular polarizable head coil (Clinical MR solutions, Brookfield, WI, USA). All images were obtained using the *T*_2_-weighted imaging method by the multi-spin-echo pulse sequence technique, with *T*_*E*_ values of 12 ms, 24 ms, 36 ms, and 48 ms, *T*_*R*_ = 3000 ms, 3 mm slice thickness, 2.5 × 2.5 cm field of view, and matrix size of 256 × 256. MR image signal intensity was measured using the signal intensity of region of interest (ROI) at different times (0, 1, 5, 10, 15, and 20 h) after injection of both synthesized and commercially available nanosized probe agents using the Dicom Works version 1.3.5 (Dicom-Works, Lyon, France) by use of the following equation:

(1) $$\begin{array}{c} \left(S=S_{0}e^{-T_{E}/T_{2}}\right),\; \end{array}$$

where *S* is the signal intensity after administration of nanoparticle agent and *S*_0_ is the signal intensity when no nanoparticle agent is applied.

### 2.8. Biodistribution Studies

To investigate the biodistribution of nanosized probe MR imaging contrast agents (SPIONs-C595, Nanomag-D-SPIO, and control) in tumor and other organs 24 h after injection all mice were sacrificed, and critical organs (including tumor, lung, heart, liver, spleen and kidney) of each of the three groups were removed and their iron content was determined by Inductively Coupled Plasma Atomic Emission Spectroscopy (ICP-AES). Each ICP-AES experiment was performed at least three times after doing acid digestion procedure [10]. The percentage of iron concentration (mg) per gram of organ was obtained as biodistribution of the conjugate in the studied organs.

## 3. Results

### 3.1. Characterization

**Figure 2:**
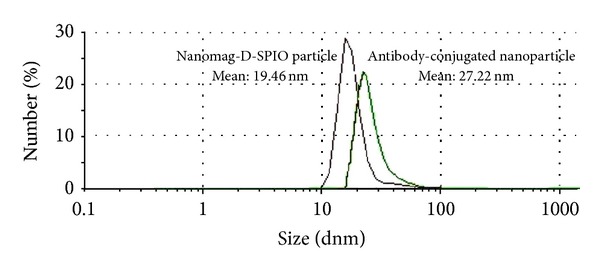
Nanoparticle size before and after conjugation.

**Figure 3:**
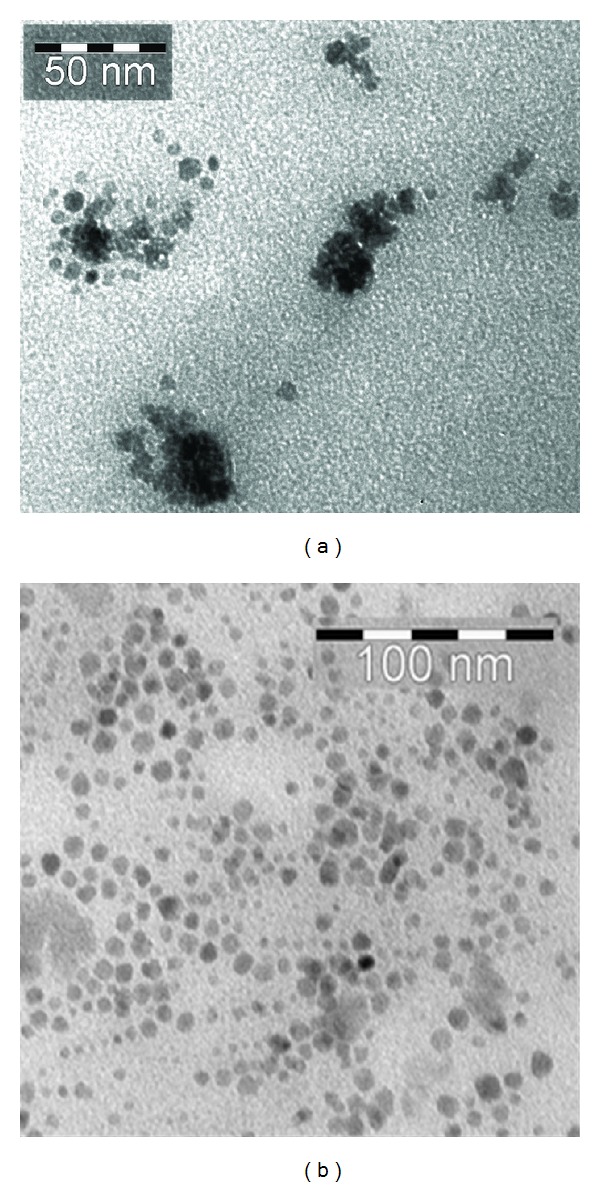
TEM images for (a) Nanomag-D-SPIO and (b) SPIONs-C595 antibody binding causes a significant reduction of particle agglomeration. The average particle size of particles estimated from TEM images was about 10–20 nm.

The particle size distribution of SPIONs before and after mAb conjugation was obtained by PCS (Figure 2). The hydrodynamic particle diameters were obtained to be 19.46 ± 0.80 nm and 27.22 ± 1.22 nm for Nanomag-D-SPIO and SPIONs-C595, respectively. Figures 3(a) and 3(b) show TEM images for the spherically shaped plain and mAb conjugated SPIONs, respectively. The average particle size calculated from TEM was 10–20 nm for two studied nanoparticle agents.

### 3.2. *In Vitro* Cytotoxicity

**Figure 4:**
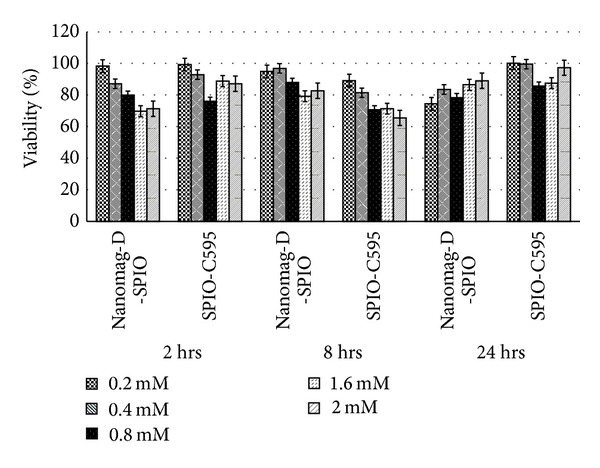
*In vitro* cytotoxicity of Nanomag-D-SPIO and SPIONs-C595 in OVCAR3 cell line by the MTT assay with different Fe concentrations ranging from 0.2 to 2.4 mM for 2, 8, and 24 h.

The *in vitro* cytotoxic effect of Nanomag-D-SPIO and the synthesized nanoprobe was assessed using the standard methyl thiazol tetrazolium bromide (MTT) assay, using ovarian cancer OVCAR3 cell line. The results after different incubation times with different iron concentrations for cell line showed more than 80% cell viability in relation to the control (Figure 4).

### 3.3. Flow Cytometry

**Figure 5:**
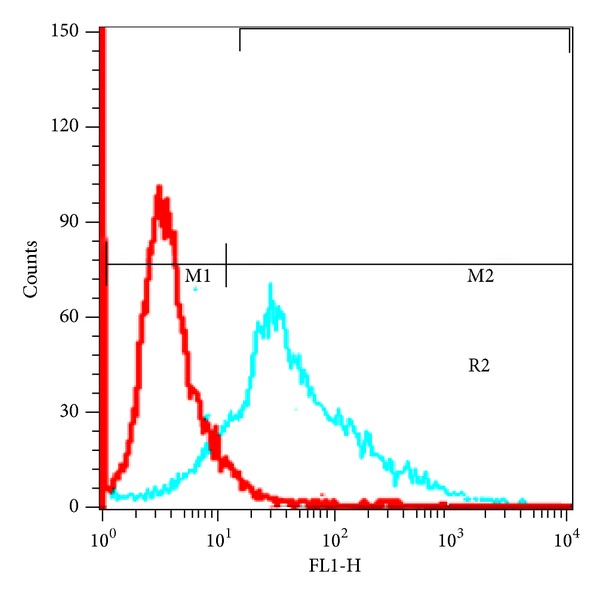
Graphs of flow cytometry for OVCAR3 cell line.

Flow cytometric analysis was performed to confirm the availability and quantitative analysis of desired ovarian cancer cell surface antigen (MUC1). Immunofluorescence staining of OVCAR3 cell line showed that OVCAR3 cell lines expresses its high levels of MUC1 on their cell surface (88.6 ± 4.6)%, as indicated in Figure 5. These results are in good agreement with a previously published study [18].

### 3.4. Cellular SPIONs Uptake

**Figure 6:**
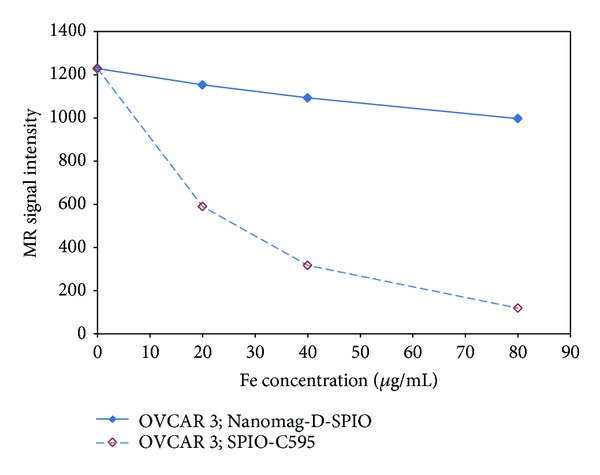
MR image signal intensity of both C595-SPIONs and Nanomag-D-SPIO at different Fe concentrations.

The capability of the synthesized nanoprobe as a specific MR imaging agent was shown in Figure 6. This figure demonstrated that the nanoprobe functionalized C595 mAb reduces 95% MR image signal intensity in OVCAR3 compared with nonspecific agent of Nanomag-D-SPIO.

### 3.5. Prussian Blue Staining

**Figure 7:**
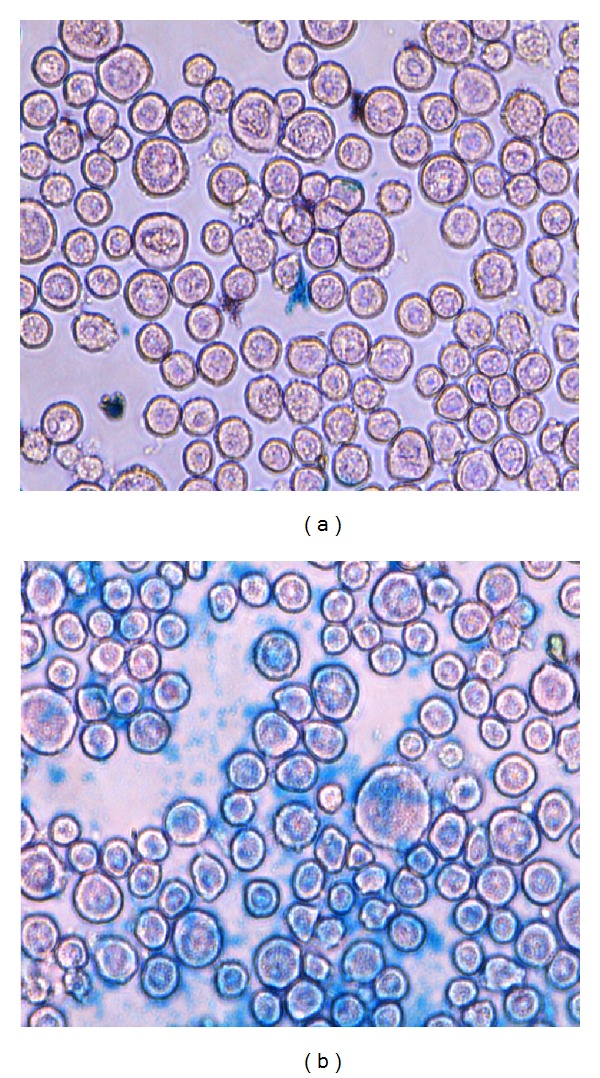
Prussian blue staining images (objective magnification: ×40) for OVCAR3 cells after 1 h incubation with (a) Nanomag-D-SPIO and (b) SPIONs-C595 nanoprobe.

The qualitative information on the cell surface antigen expression as well as specificity and cellular uptake of SPIONs-C595 and Nanomag-D-SPIO to the cells were done by Prussian blue staining test. Blue areas are shown in Figure 7. As can be seen from this figure, there was no blue color appearance in the cells incubated with nonfunctionalized particles.

### 3.6. MR Signal Intensity

**Figure 8:**
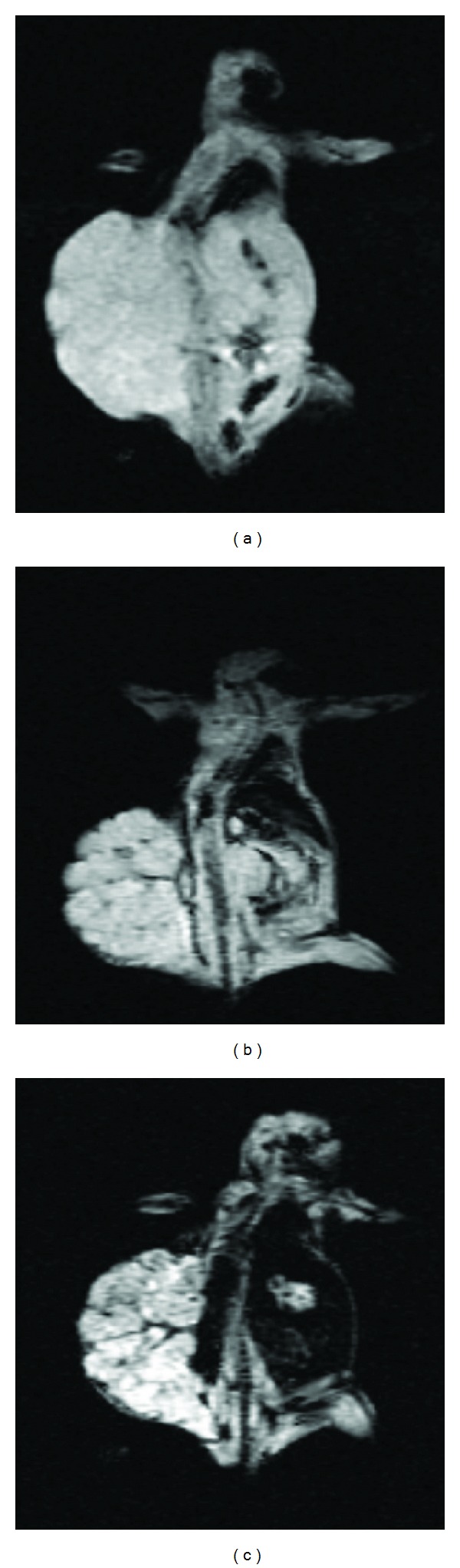
MR image of mice: (a) before injection of agents, (b) after injection of Nanomag-D-SPIO, and (c) after injection of C595-SPIONs.

**Figure 9:**
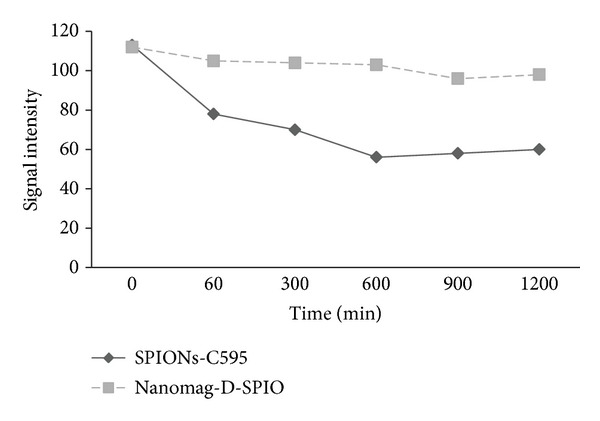
MR image signal intensity of tissues from some region of interest (ROI) at different time after injection of two studied agents.

MR images of studied animals before nanoprobe injection and after injection for both agents are shown in Figure 8. For *in vivo* MR imaging, distinct changes of signal intensities and *T*_2_ values of ovarian cancers were detected after the injection of SPIONs-C595 compared to Nanomag-D-SPIO (Figure 9).

### 3.7. Biodistribution

**Figure 10:**
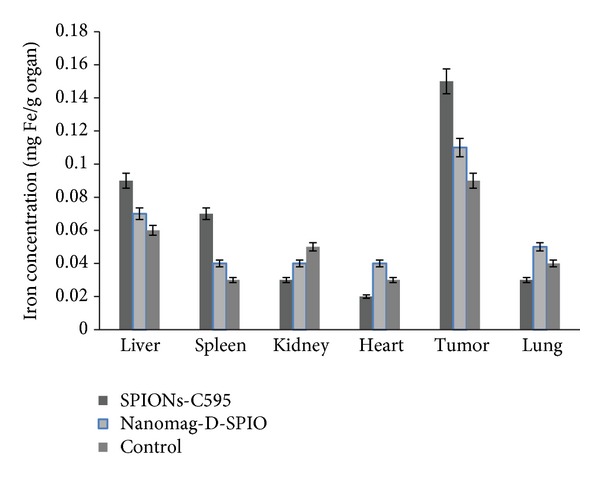
Biodistribution of iron uptakes in different studied organs 24 h after injection of nanoparticles.

The percentage of injected dose per gram of each organ was measured by ICP-AES test, shown in Figure 10. It showed that most of the injected dose was found in tumor. Liver was the second organ which attained SPIONs after 24 h postinjection, and its clearance is so fast in other organs. 

## 4. Discussion

The most advantage of molecular imaging such as MR imaging and Positron Emission Tomography (PET) is the kinetic analysis of a given molecular event in the same experimental subject over time [18, 19]. The potential broad applications of molecular imaging, in particular MR imaging, events *in vivo* lie in the study of cell biology, signal enhancement, and early stage tumor detection. Most importantly, MR imaging will have great implications for the identification of potential nanoparticle contrast agents. The main disadvantages of MR imaging contrast agents are related to a lower cellular uptake and nonspecific contrast agent targeting. As a result, it is delicious to generate techniques for delivering agents with cost benefit paramagnetic metals such as SPIONs for early tumor detection. The present investigation highlights the pharmacokinetic behavior of SPIONs conjugated C595 mAb, including organ distribution and MR image signal enhancement.

Both results of *in vitro* cytotoxicity and flow cytometry (Figures 4 and 5) showed that OVCAR3 cell lines expresses its high levels of MUC1 on their cell surface. These results are in good agreement with previously published results [16] which also showed a high affinity of SPIONs-C595 for OVCAR3 cell line. 

The capability of the synthesized nanoprobe as a specific MR imaging contrast agent was shown in Figure 6. This figure demonstrated that the nanoprobe functionalized C595 mAb reduces 95% MR image signal intensity in OVCAR3 compared with nonspecific agent of nanomag-D-SPIO.

Prussian blue staining results (Figure 7), illustrated the targeting effect and SPIONs uptake of functionalized particles (SPIONs-C595) on the cellular uptake behavior. 

MR images of studied animals which are shown in Figure 9, demonstrated that the reduction of signal intensity were 56% and 10% for SPIONs-C595 and Nanomag-D-SPIO, respectively. The results of biodistribution study suggested that SPIONs-C595 may be potentially used as nanoparticle contrast agent in MR imaging. The conjugated nanoparticles affinity for OVCAR3 cell lines was five times higher than that of non-conjugated nanoparticles. Both MR imaging and ICP-AES results showed significant preferential uptake of the SPIONs-C595 nanoparticles by human ovarian cells (OVCAR3) as compared to the other studied organs (Figure 10). *In vivo* results also showed that tumor uptake of SPIONs-C595 was about two times higher than the other organs. The biodistribution of nanosized agents in mice showed dramatic uptake in reticuloendothelial system after 24 h postinjection, and this may be due to their large particle size. Of course, the physiochemical properties of synthesized SPIONs such as coating, particle size, and morphology are important in determining their *in vivo* distribution [20].

Moreover, due to significant uptakes of nanoparticles in liver and spleen and their fast clearance from other organs, these results suggest that this nanosized probe could be suitable for use as target agents for the detection of liver and spleen tumors.

The present results are essential for developing SPIONs as a potential contrast agent delivery technique for ovarian cancer. Such a technique will facilitate the use of multimodality imaging techniques, including ultrasound, PET, and single positron emission computed tomography (SPECT) hence improving the ability and the efficacy of early diagnosis.

## 5. Conclusions

The novel MR imaging nanosized probe used in this study was prepared with an mAb C595 conjugated to SPIONs to target the MUC1 receptor expressed by most of ovarian cancer cells.

Findings of the present study showed that, functionalization of SPIONs-C595 to the MUC1-expressing cells is achievable both *in vitro* and *in vivo*. The results of flow cytometry, Prussian blue staining, signal intensity, biodistribution and measurement of iron uptake by cells showed high targeting and affinity of SPIONs-C595 to MUC1 positive ovarian cancer cells (OVCAR3). Overall, results demonstrated high sensitivity of synthesized nanoprobe as MR imaging contrast agent for the detection of ovarian cancer cells. Of course, more study is needed for confirming the results using of PET as an alternative method of molecular imaging.
